# Regional Differences and Influencing Factors of Allocation Efficiency of Rural Public Health Resources in China

**DOI:** 10.3390/healthcare8030270

**Published:** 2020-08-14

**Authors:** Tao Liu, Jixia Li, Juan Chen, Shaolei Yang

**Affiliations:** 1 School of Finance and Economics, Henan Polytechnic University, Jiaozuo 454000, China; liutao2511001@hpu.edu.cn; 2 School of Emergency Management, Henan Polytechnic University, Jiaozuo 454000, China; lijixiahpu@163.com; 3 School of Economics and Management, Southwest Petroleum University, Chengdu 610500, China; chenjuan@swpu.edu.cn; 4 Chinese Studies Center, Sichuan University, Chengdu 610065, China

**Keywords:** allocation efficiency of rural public health resources, game cross-efficiency model, theil index, gini index, bootstrap truncated regression model

## Abstract

In the face of increasingly growing health demands and the impact of various public health emergencies, it is of great significance to study the regional differences in the allocation efficiency of the rural public health resources and its improvement mechanism. In this paper, the game competition relationship is included in the evaluation model, and the game cross-efficiency model is used to measure the allocation efficiency of the rural public health resources in 31 provinces of China from 2008 to 2017. Then, the Theil index model and the Gini index model are applied in exploring the regional differences in the allocation efficiency of rural public health resources and its sources. Finally, the bootstrap truncated regression model is used to analyze the influencing factors of the allocation efficiency of the rural public health resources in China. The results show that, first, the total allocation efficiency level of the rural public health resources in China from 2008 to 2017 is relatively low, and it presents a U-shaped trend, first falling and then rising. Second, the changing trend of the allocation efficiency of the rural public health resources in the eastern, central, and western regions of China is similar to that in the nationwide region, and it shows a gradient trend that “the allocation efficiency in the eastern region is high, the allocation efficiency in the western region is low, and the allocation efficiency in the Central region is at the medium level”. However, the gap among the three regions is continually narrowing. Third, the calculation results of the Theil index and the Gini index show that intra-regional differences are the major source of the regional differences in the allocation efficiency of the rural public health resources in China, and the inter-regional differences demonstrate an expansion trend. Finally, the improvement of the education level and the social support level will generally improve the allocation efficiency of the rural public health resources in China and its three regions. The increased governmental financial support and urbanization level will reduce the allocation efficiency of the rural public health resources in China and its three regions. The economic development level, the living conditions and the population density are the important influencing factors of the allocation efficiency differences of the rural public health resources in the three regions. Therefore, on the basis of ensuring the increase of the total supply of the rural public health resources, more attention should be paid to the improvement of the allocation efficiency. Moreover, on the basis of continually narrowing the inter-regional differences among the eastern, central, and western regions, more attention should be paid to the intra-regional differences of the allocation efficiency of the rural public health resources among the different provinces. The various economic and social policies should be constantly optimized to jointly improve the allocation efficiency of the rural public health resources.

## 1. Introduction

People’s health is an important symbol of national prosperity. With the rapid development of China’s economy, the people’s demand for health services is growing continually. To meet the demand, there must be a high-quality public health service system. As a developing country, China has a large rural population. The data from National Bureau of Statistics of the People’s Republic of China indicates that the rural population of the Chinese mainland was 564 million at the end of 2018, accounting for 40.42% of the total population. However, the per capita health expenditure of the rural residents was only 2477 yuan, accounting for only 41.31% of the per capita health expenditure of the urban residents. Several main indexes reflecting the public health situation in the rural areas, such as the total service amount of the public health, the diagnosis and treatment person-time, the utilization rate of hospital beds, and the number of beds in the township hospitals per 1000 person, showed a downward trend [1]. According to the statistical information of National Health Commission of the People’s Republic of China, in recent years, with the continuous deepening of the poverty alleviation strategy, the proportion of poverty caused by diseases has not decreased, but increased from 42.2% in 2013 to 44.1% in 2015. This shows that diseases have become one of the main reasons for the increase of poverty [2]. The economist Banerjee won the Nobel Prize in Economics in 2019 for his contributions to global poverty alleviation through experimental methods. His research found that investment in the health of rural poor groups can improve their health level and then reduce the poverty caused by diseases [3]. Therefore, in consideration of the current urban and rural public health resource situation and the poverty alleviation, the rural public health career must be the top priority of the whole public health career development and therefore great importance should be attached to this. In addition, owing to the change of climate and environment as well as the increasingly frequent cross-border movements, the spread of infectious diseases has become ever more serious, such as the frequent occurrence of influenza including H1N1 and H7N9 in recent years and the attack of “dengue fever”. In particular, the corona virus disease 2019 (COVID-19) incident, which began at the end of 2019, has brought a severe challenge to the rural public health service system of China. In the face of the increasingly growing health demand of the people and the impact of various public health emergencies, it is of great significance to study how to improve the allocation efficiency of the rural public health resources and ensure the effective supply of the rural public health resources.

Public health has always been the focus of attention in countries around the world. How to improve the effective allocation of the public health resources is the major problem facing most countries in the world [4]. At the same time, it has also attracted the extensive attention from academia, and a large number of studies of the effective allocation of the public health resources have been carried out. Some scholars have discussed the evaluation method of the hospital efficiency. For example, Varabyova et al. [5] and Xu et al. [6] comparatively analyzed the application of ratio analysis (RA), stochastic frontier analysis (SFA), and data envelopment analysis (DEA) in the hospital efficiency evaluation. Mitropoulos et al. [7] and Rouyendegh et al. [8] respectively combined the DEA method with Bayesian analysis and fuzzy analytic hierarchy process (FAHP) method to evaluate the hospital efficiency. Due to the complexity of the public health resource supply, a single index cannot fully reflect its allocation efficiency. The DEA method can be used to evaluate multiple input and output indexes, has become the first choice for scholars. At present, many scholars use the DEA method to analyze the allocation efficiency of the public health resources from different angles, mainly including the following two aspects below.

First, the hospital efficiency in different countries or regions is discussed by using the classical DEA method from the microcosmic level. Kawaguchi et al. [9], Sohn et al. [10], Chowdhury et al. [11], Gholami et al. [12], Flokou et al. [13], Blatnik et al. [14], Campanella et al. [15], and Fuentes et al. [16] respectively evaluated the hospital efficiency of developed countries or regions, namely Japan, South Korea, the United States, Ontario, Greece, Slovenia, Italy, and Murcia of Spain. Jat et al. [17] and Gimenez et al. [18] assessed the hospital efficiency of the developing countries India and Colombia. Other scholars have evaluated the hospital efficiency in China and some areas. Hu et al. [19] used the undesirable output DEA method to evaluate China’s regional hospital efficiency. Cheng et al. estimated the efficiency of 48 rural township hospital in Xiaogan city of Hubei province, China from 2008 to 2014 [20]. Zheng et al. evaluated the relative efficiency of the public hospitals in China after the implementation of new medical reforms [21]. Li et al. analyzed the determinants and differences of the township hospital efficiency among Chinese provinces from 2003 to 2016 [22]. Other scholars have further discussed the impacts of management and organization [23], the medical reform [24,25], and the increasing geographic elevation [26] on hospital efficiency.

Second, the classical DEA method is used to study the allocation efficiency of the public health resources among different countries and within a country from the macroscopic level. Some scholars have evaluated and studied the efficiency of the public health systems in 171 countries worldwide [27], the organization for economic co-operation and development (OECD) countries [28,29], the low- and middle-income countries [30], and the Asian countries [31]. Other scholars have estimated the public health efficiency in Greece [32], India [33], Lebanon [34], México [35], and Slovakia [36]. Some scholars have deeply discussed China’s public health efficiency, and respectively calculated and studied the Chinese provincial community health service efficiency [37,38,39,40,41,42] and the allocation efficiency of the public health resources in the coastal provinces of China [43]. A few scholars have preliminarily analyzed the allocation efficiency of the rural public health resources [44,45]. After analyzing the allocation efficiency of the public health resources, some scholars further discussed the influencing factors. Mitropoulos et al. and Lee et al. assessed the impact of the public health policies on the health efficiency [46,47]. Han et al. [48] introduced such variables as population density, per capita gross domestic product (GDP), the residents’ education level, the fiscal decentralization, and the healthcare system reform into the Tobit model. Zhang [49] and Liu [50] incorporated fiscal decentralization, the medical and health system reform policies, per capita GDP, the residents’ education level, the population density, and the urbanization level into the explained variables. Guo et al. believe that the social, economic, and policy variables, such as the population density, the residents’ education level, and the fiscal decentralization, are important reasons for the efficiency difference [51].

To sum up, the research results of scholars such as Kawaguchi [9], Jat [17], Li [22], Liu [37], and Xue [45] on the measurement of the allocation efficiency of the public health resources and its influencing factors provide a great deal of experience as a reference for the study of this paper. Compared with the existing studies, this paper has three main contributions.

First, a comprehensive and systematic study on the regional differences and the causes of the allocation efficiency of the rural public health resources in China is conducted in this paper. Although the existing studies cover multiple levels, they are less involved in the field of the rural public health, and there are even fewer studies that explore it from the perspective of regional differences. Second, the game competition relationship is included in the evaluation model, and the improved game cross-efficiency model is used to replace the traditional DEA model. This solves the problem of overestimating the allocation efficiency of the regional public health resources in the traditional DEA model. Third, when analyzing the influencing factors of the allocation efficiency of the rural public health resources, the traditional Tobit regression model is replaced by the bootstrap truncated regression model. This solves the deviation problem of the classical Tobit regression model when measuring the influencing factors of efficiency [52].

Consequently, this paper uses the game cross-efficiency model and Theil index model to evaluate and analyze the regional differences and the causes of the allocation efficiency of the rural public health resources in 31 provinces of China from 2008 to 2017, and uses the bootstrap truncated regression model to find out the influencing factors, so as to provide the policy basis for improving the allocation efficiency of the rural public health resources in China.

## 2. Method and Data

### 2.1. Game Cross-Efficiency Model

The game cross-efficiency model is an improvement to the traditional DEA model. In the evaluation process of the traditional DEA models, such as the Charnes-Cooper-Rhodes (CCR) model [53] and the Banker-Charnes-Cooper (BCC) model [54], each decision making unit (DMU) tends to give more weight to itself so as to result in the overestimation of its efficiency. In order to overcome this shortcoming, Sexton proposed a cross-efficiency DEA model [55]. Based on the CCR model framework, the weight of mutual evaluation was added between DMUs to correct the pure self-evaluation problem in the traditional CCR. However, as CCR and BCC models have more than one optimal weight, the cross-efficiency value is not unique. In order to solve this problem, Liang et al. proposed a game cross-efficiency model. On the basis of solving the problem that the traditional CCR and BCC models cannot be effectively ordered, the game relationship between the evaluation units is introduced. While avoiding the secondary target selection of the cross-efficiency model, the strict assumption conditions of the traditional models are relaxed to make it more practical [56,57].

The main operation process of the model is as follows: assume that there are *n* DMU, and each decision-making unit ${DMU}_{j}$ obtains *s* outputs through *m* inputs. The *i* input and the *r* output of $DMU_{j}\left(j=1,\cdots ,n\right)$ is expressed, respectively, as $x_{ij}\left(i=1,\cdots ,m\right)$ and $y_{rj}\left(r=1,\ldots ,s\right)$ . First, the efficiency value of any evaluation unit $DMU_{d}$ under the CCR model is obtained by solving the following linear programming problem:

(1) $$\begin{array}{l} maxE_{dd}=\sum_{r=1}^{s}\mu_{rd}y_{rd}\\ s.t.\sum_{i=1}^{m}\omega_{id}x_{id}=1\\ \sum_{r=1}^{s}\mu_{rd}y_{rj}-\sum_{i=1}^{m}\omega_{id}x_{ij}\leq 0,j=1,2,\ldots ,n\\ \mu_{rd^{’}}\omega_{id}\geq 0,r=1,2,\ldots ,s;i=1,2,\ldots ,m. \end{array}$$

In Equation (1), $\omega_{id}$ and $\mu_{rd}$ are respectively the *i* input weight and the *r* output weight of the evaluation unit $DMU_{d}$. Second, Equation (1) is used to solve the cross-efficiency $E_{dj}$ of ${DMU}_{j}$ taking $DMU_{d}$ as its weight:

(2) $$E_{dj}=\frac{\sum_{r=1}^{s}\mu_{rd}^{*}y_{rj}}{\sum_{i=1}^{m}\omega_{id}^{*}x_{ij}},d,j=1,2,\ldots ,n$$

By solving Equation (2), the *n* sets of optimal weights $\omega_{1d}^{*},\omega_{1d}^{*},\ldots ,\omega_{md}^{*}$ and $\mu_{1d}^{*},\mu_{1d}^{*},\ldots ,\mu_{sd}^{*}$ can be obtained. Then, all results of the cross-efficiency $E_{dj}$ constitute the following cross-efficiency matrix:

$$E=\left[\begin{array}{cccc} E_{11} & E_{12} & \ldots & E_{1n}\\ E_{21} & E_{22} & \ldots & E_{2n}\\ \ldots & \ldots & \ldots \\ E_{n1} & E_{n1} & \ldots & E_{nn} \end{array}\right]$$

Therein, the elements on the main diagonal, $E_{dd},d=1,\cdots n$, are the optimum solution of the CCR model, namely the self-evaluation efficiency value of the traditional DEA model. The elements on the off-diagonal are the cross-efficiency value that the decision-making unit $DMU_{j}(j=1,\cdots ,n,and\;j\neq d)$ obtains by using the weight of $DMU_{d}$ . Then, the cross-efficiency value of the decision-making unit $DMU_{j}\left(j=1,\cdots ,n\right)$ is the arithmetic mean value of the corresponding j column in the matrix:

(3) $$\bar{E}=\frac{1}{n}\sum_{d=1}^{n}E_{dj}$$

It should be noticed that the optimal weight of Equation (1) is not unique, and accordingly, the cross-efficiency value of the decision-making unit $DMU_{d}$ taking $DMU_{d}$ as its weight is not unique either. The final cross-efficiency is determined in the multiple optimum solutions by introducing the quadratic objective. Meanwhile, because there is a direct or indirect competitive relation between each decision-making unit, the final efficiency value can be determined by game. It is assumed that there is a non-cooperative game relationship between participants and this relationship is reflected in the constraint conditions of the mathematical programming. Suppose that the efficiency value of the participant $DMU_{d}$ is $\alpha_{d}$, and the remaining participant $DMU_{j}$ maximizes its own efficiency value while keeping the efficiency value of $DMU_{d}$ from being reduced. Here, the game cross-efficiency value that $DMU_{j}$ obtains by using the weight of $DMU_{d}$ is defined as:

(4) $$\alpha_{dj}=\sum_{r=1}^{s}\mu_{rj}^{d}y_{rj}/\sum_{i=1}^{m}\omega_{ij}^{d}x_{ij}d,j=1,\cdots ,n$$

In Equation (4), $\mu_{rj}^{d}$ and $\omega_{ij}^{d}$ are the feasible weights of the model, while $\alpha_{dj}$ is the game cross-efficiency of $DMU_{j}$ for $DMU_{d}$, and can be calculated by the following linear programming:

(5) $$\begin{array}{l} Max\sum_{r=1}^{s}\mu_{rj}^{d}y_{rj}\\ s.t.\sum_{i=1}^{m}\omega_{ij}^{d}x_{ij}-\sum_{r=1}^{s}\mu_{rj}^{d}y_{rj}\geq 0,j=1,\cdots ,n\\ \sum_{i=1}^{m}\omega_{ij}^{d}x_{ij}=1\\ \alpha_{d}\times \sum_{i=1}^{m}\omega_{ij}^{d}x_{id}-\sum_{r=1}^{s}\mu_{rj}^{d}y_{rj}\leq 0\\ \omega_{ij}^{d}\geq 0,i=1,2,\cdots ,m\\ \mu_{rj}^{d}\geq 0,r=1,2,\cdots ,s \end{array}$$

In Equation (5), $\alpha_{d}\leq 1$ is the parameter. Its initial value is the traditional cross-efficiency value, and its subsequent value can be calculated through the iterative algorithm. In summary, the game cross-efficiency value of $DMU_{j}$ is defined as:

(6) $$\bar{\alpha_{j}}=\frac{1}{n}\sum_{d=1}^{n}\alpha_{dj}$$

This paper applies the advanced MaxDEA UItra8.0 software to solve the complex linear programming problem in the game cross-efficiency model.

### 2.2. Theil Index Model

Theil index model was originally proposed by Theil to measure the differences between samples, and can effectively measure the contribution of the intra- and inter-group gaps to the total gap [58]. This paper uses Theil index model to measure the regional gap of the rural public health resource allocation efficiency in China. Because of the additivity of Theil index, the total regional differences are decomposed into the intra-regional differences and the inter-regional differences.

First of all, the total regional differences of the allocation efficiency of the rural public health resources are measured by the total Theil index (TL), and the methods of decomposing the Theil index and its structure by Bourguignon, Cowell, and Shorrocks are used for reference [59,60,61]. Thus, the calculation formula is $TL=\frac{1}{n}\sum_{i=1}^{n}\frac{y_{i}}{\bar{y}}log\left(\frac{y_{i}}{\bar{y}}\right)$. The intra-regional differences are measured by the intra-regional Theil index, and the calculation formula is ${TL}_{w}=\sum_{k=1}^{m}\left(\frac{n_{k}}{n}\frac{\bar{y_{k}}}{\bar{y}}\right){TL}_{k}$. The inter-regional differences are measured by the inter-regional Theil index, and the calculation formula is ${TL}_{b}=\sum_{k=1}^{m}\frac{n_{k}}{n}\left(\frac{\bar{y_{k}}}{\bar{y}}\right)\log\left(\frac{\bar{y_{k}}}{\bar{y}}\right)$. In the above formulas, y represents the allocation efficiency of the rural public health resources in each province, *n* represents the number of provinces, *n_k_* represents the number of provinces in *k* region. In addition, the ratio of the intra-regional Theil index to the total Theil index, namely, *TL_w_/TL*, represents the contribution rate of the intra-regional differences to the total regional differences. Similarly, the ratio of the inter-regional Theil index to the total Theil index, namely *TL_b_/TL* represents the contribution rate of the inter-regional differences to the total regional differences.

### 2.3. Gini Index Model

The Gini index Model proposed by Dagum (1997) [62] is used to analyze the differences in the allocation efficiency of the rural public health resources in China and its three regions. According to the Gini index and its subgroup decomposition method proposed by Dagum, the Gini coefficient of the allocation efficiency of the rural public health resources in China can be defined as:

(7) $$G=\sum_{h=1}^{k}\sum_{j=1}^{k}\sum_{i=1}^{n_{k}}\sum_{r=1}^{n_{j}}|y_{hi}-y_{jr}|/2n^{2}\bar{y}$$

Thereinto, $y_{hi}$ ($y_{\mathrm{jr}}$) is the allocation efficiency of the rural public health resources in *h*(*j*) region, $\bar{y}$ is the mean value of the allocation efficiency of the rural public health resources in each region, *n* is the number of provinces, *k* is the number of regions, $n_{h}$ ($n_{k}$) is the number of provinces in *h*(*j*) region, *G* is the total Gini index, *h* and *j* are the different region division, and *i* and *r* are the different provinces in the region. According to the Gini index decomposition method proposed by Dagum, *G* = *G_w_* + *G_nb_* + *G_t_*. The regional difference of the allocation efficiency of the rural public health resources can be accordingly divided into three parts: *G_w_* represents the intra-regional difference contribution of the total differences of the allocation efficiency of the rural public health resources, *G_nb_* represents the inter-regional difference contribution of the total differences of the allocation efficiency of the rural public health resources, and *G_t_* represents the contribution of the intensity of transvariation of the inter-regional allocation efficiency of the rural public health resources. The specific calculation formula can be seen in the literature of Dagum [62].

### 2.4. Bootstrap Truncated Regression Model 

The value range of the allocation efficiency of the rural public health resources is (0, 1], and it belongs to the truncated data. If the least squares method is directly used for the regression analysis, the results will be biased and inconsistent. Simar and Wilson proved that the classic Tobit regression model for processing the truncated data is not suitable for testing the influencing factors of efficiency, and accordingly proposed the bootstrap truncated regression model that can minimize the uncertainty of data and the statistical noise to overcome this limitation [52]. The expression is as follows:

(8) $$\theta_{i}=Z_{i}\beta +\varepsilon_{i}$$

In the Equation (8), $\theta_{i}$ is the explained variable, β is the regression parameter, $Z_{i}$ is the explanatory variable, and $\varepsilon_{i}$ obeys the normal distribution of $N(0,\delta^{2})$, *i* = 1, 2 ,…, *n*.

### 2.5. Index System and Data Sources

(1) Input index: The input index of the public health resources usually includes three main categories, that is, the health human resources, the health material resources and the health financial resources. In the design of the specific indexes, the number of doctors, nurses and beds are generally selected as the input indexes [17,25]. According to the statistical data of the health departments in China, considering the representativeness and accessibility of the input index, the number of personnel in the rural health institutions (the total number of doctors and nurses) is selected as an alternative index of the labor input, and the number of beds in the rural health institutions is selected as an alternative index of the material input. Meanwhile, considering the fact that health institutions are the important spatial carrier for carrying out the health activities, the number of the rural health institutions is also used as another alternative index of the material input. Although drugs are an important variable of the material input, they are mainly suitable for the hospital efficiency evaluation level. Because it is difficult to obtain the regional data of drugs, they are not considered here. The rural medical and healthcare expenditure can provide the financial support for the rural health activities, and so it is selected as an alternative index of the financial input.
(2) Output index: The final output of the public health resource input is the improvement of the population health. However, because of the complexity of the health improvement measurement and the difficulty of the data acquisition, some process indexes are usually used to replace it [5]. According to the statistical data of the health departments in China, considering the representativeness and accessibility of the output index, the rural diagnosis and treatment person-time, the rural number of people receiving hospitalizations and the rural average hospitalization days are selected as the output indexes of the rural public health resources. See Table 1 for details.

**Figure 1:**
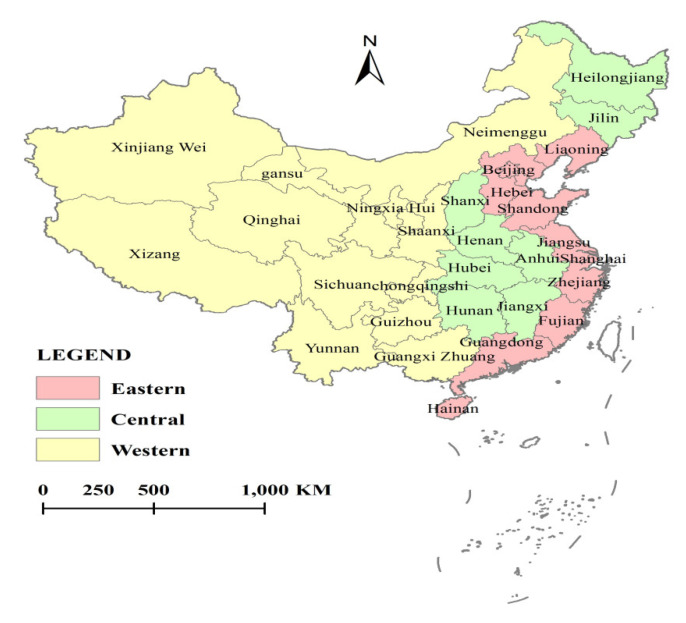
Distribution map of Eastern, Central and Western regions in China.

The evaluation object of this paper is 31 provinces in Chinese Mainland except Hong Kong, Macao, and Taiwan. According to the administrative division of Chinese Mainland, the 31 provinces are divided into the eastern, central, and western regions. The eastern region includes 11 provinces, namely, Beijing, Tianjin, Hebei, Liaoning, Shanghai, Jiangsu, Zhejiang, Fujian, Shandong, Guangdong, and Hainan. The central region includes 8 provinces, namely, Shanxi, Jilin, Heilongjiang, Anhui, Jiangxi, Henan, Hubei, and Hunan. The western region includes 12 provinces, namely, Sichuan, Chongqing, Guizhou, Yunnan, Tibet, Shaanxi, Gansu, Qinghai, Ningxia, Xinjiang, Guangxi, Inner Mongolia. They are shown in Figure 1. According to the study purpose and the accessibility of data, this paper selects 2008–2017 as the investigation years, and collects the panel data containing 31 truncated units within 10 years, with a total of 310 observation samples. The data in this paper comes from the hygiene and health statistical yearbook of China (2009–2018), the population and employment statistical yearbook of China (2009–2018) and the rural statistical yearbook of China (2009–2018), and the panel data of 31 provinces in China from 2008 to 2017 are finally collated and summarized.

## 3. Results and Discussion

### 3.1. Allocation Efficiency of Rural Public Health Resources in China: Comparison between CCR Model and Game Cross-Efficiency Model

**Figure 2:**
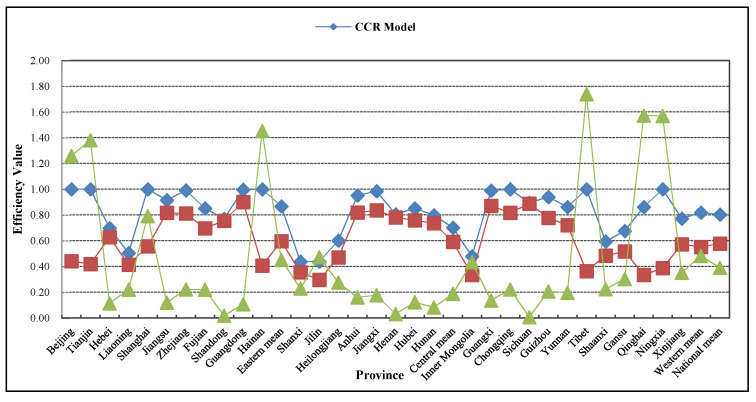
Comparison of allocation efficiency of rural public health resources in each province of China from 2008 to 2017: CCR model and game cross-efficiency model.

This paper applies MaxDEA UItra8.0 software and selects the CCR model and the game cross-efficiency model, and measures the average situation of the rural public health resource allocation efficiency of 31 provinces in China from 2008 to 2017 under the two DEA models, and calculates the efficiency variance value of the two models, as shown in Figure 2.

Through the comparison between the CCR model and the game cross-efficiency model, it can be found that the efficiency value measured by the CCR model is obviously higher than that of the game cross-efficiency model. From the perspective of the national level, the efficiency value (0.804) measured by the CCR model is higher than that (0.578) of the game cross-efficiency model, which is 28.1% higher on average. From the perspective of the eastern, central, and western regions, the efficiency value measured by the CCR model is respectively 0.868, 0.703, and 0.820 and is higher than that (0.597, 0.592, and 0.553) measured by the game cross-efficiency model, which is 31.2%, 15.8%, and 32.6% higher, respectively. From the perspective of each province, the efficiency value measured by the CCR model is higher than that of the game cross-efficiency model, and the higher range is slightly different. This paper uses the deviation to measure the range that the CCR model is higher than the game cross-efficiency model. As shown in Figure 2, the deviation in Tibet is the highest, and the efficiency value measured by the CCR model is 1. However, after it is proofread by the game cross-efficiency model, the actual efficiency value is only 0.365 and the deviation is as high as 63.5%.

Generally speaking, if the game relationship between each region is not taken into consideration, the measured efficiency value of the rural public health resource allocation in each province will be exaggerated, and is not consistent with the actual situation of the rural public health resource allocation. In order to solve this problem, this paper uses the game cross-efficiency model to measure the allocation efficiency of the rural public health resources in 31 provinces in China, and truly reveals the actual situation of the allocation efficiency of the rural public health resources in each province of China. Next, the game cross-efficiency model will be used to analyze the allocation efficiency situation of the rural public health resources in China in detail.

### 3.2. Temporal and Spatial Evolution of Allocation Efficiency of Rural Public health Resources in China

#### 3.2.1. Interannual Changes

**Figure 3:**
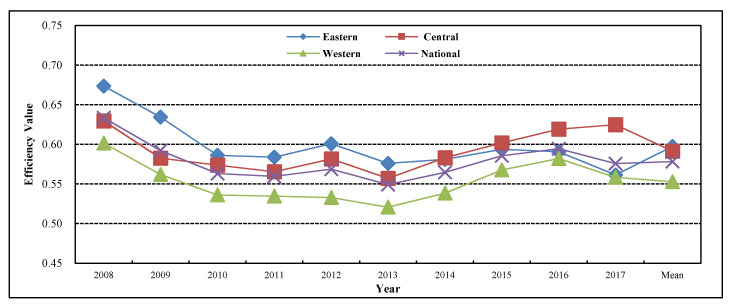
Allocation efficiency of rural public health resources in China and its Eastern, Central and Western regions from 2008 to 2017.

Figure 3 shows the interannual change situation of the allocation efficiency of the rural public health resources in China and its eastern, central, and western regions from 2008 to 2017.

From the perspective of the overall national situation, the total level of the allocation efficiency of the rural public health resources in China from 2008 to 2017 is relatively low, and presents a U-shaped trend of first falling and then rising. This conclusion is similar to the research results of Liu et al. [37]. The average value of the allocation efficiency of the rural public health resources in China from 2008 to 2017 is 0.578, and is still far from the efficient frontier. This indicates that the allocation level of the rural public health resources in China needs to be improved and the utilization efficiency of the rural public health resources is low. In 2008, the allocation efficiency of the rural public health resources was 0.634; it was in a fluctuating state of decline from 2009 to 2013, and had fallen to 0.549 by 2013. This is mainly because in 2009, China made an important strategic deployment of deepening the medical and health system reform, established the new rural cooperative medical insurance as the rural basic medical security system, and gradually improved the subsidy standard of every level of the government finance for the new rural cooperative medical insurance. The governmental subsidy standard for the new rural cooperative medical insurance was improved from 120 yuan per person per year in 2010 to 200 yuan per person per year in 2011, from 200 yuan per person per year to 240 yuan per person per year in 2012, and from 240 yuan per person per year to 280 yuan per person per year in 2013. A large number of investments in the rural public health fund are not fully utilized, which leads to the decline of the allocation level of the rural public health resources. With the new round of the medical and health system reform, it had been in a sustained rising state since 2013 and had risen to 0.576 by 2017. This shows that the medical and health system reform has entered a stable period and the policy effect is beginning to gradually appear. This conclusion is similar to the research results of Du et al. [42].

From the perspective of the three regions, the changing trend of the allocation efficiency of the rural public health resources in the eastern, central, and western regions of China from 2008 to 2017 is similar to that in the nationwide region. In 2008, the allocation efficiency of the rural public health resources in the eastern, central, and western regions was respectively 0.674, 0.630, and 0.602 and then tended to decline, and had fallen to a lower level by 2013. It had been in a sustained rising state since 2013 and presents a U-shaped trend of first falling and then rising as a whole.

From the perspective of the regional comparison, it shows a gradient trend that “the allocation efficiency in the eastern region is high, the allocation efficiency in the western region is low, and the allocation efficiency in the central region is at the medium level”, and this conclusion is similar to the research results of Jiang et al. [25]. However, the gap among the three regions is continually narrowing. The efficiency value in the eastern, central, and western regions from 2008 to 2017 is respectively 0.597, 0.592, and 0.553, and presents a state that “the efficiency value in the eastern region is the highest, the efficiency value in the western region is the lowest, and the efficiency value in the central region is at the medium level” as a whole.

From the perspective of different years, the gap among regions is continually narrowing. In 2008, the eastern region with the highest efficiency value was 0.072 higher than the western region with the lowest efficiency value. The gap between the two regions had been continually narrowing since then, and the eastern region was only 0.003 higher than the western region in 2017.

#### 3.2.2. Interprovincial Changes

**Figure 4:**
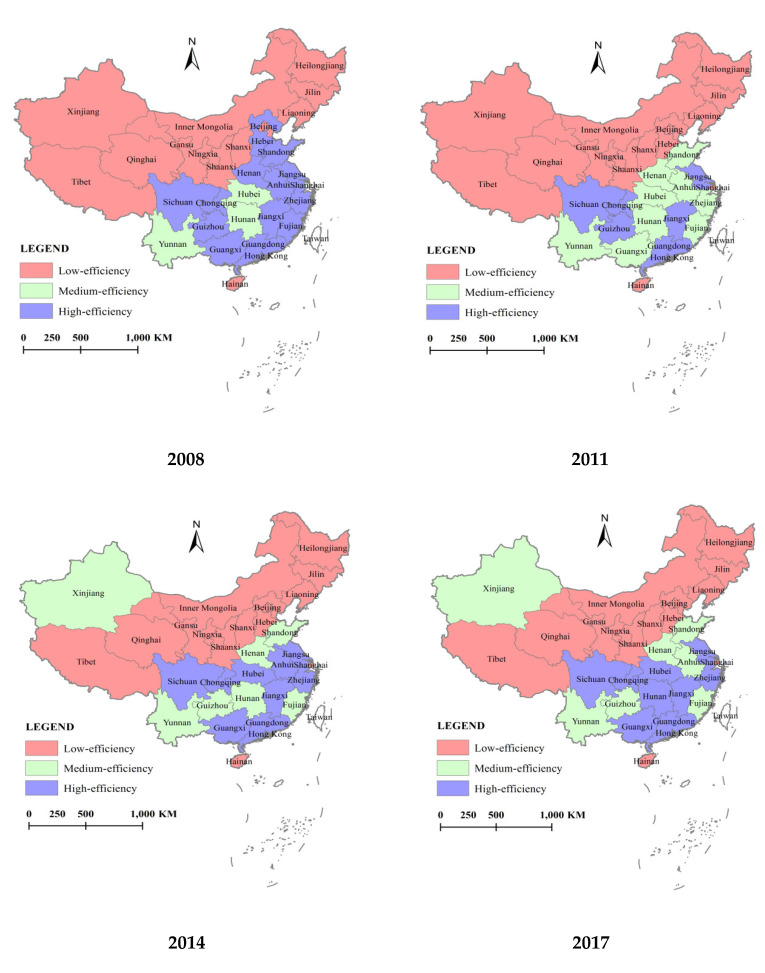
Spatial distribution of allocation efficiency of rural public health resources in each province of China from 2008 to 2017.

The allocation efficiency value of the rural public health resources (AEV) in 31 provinces of China is divided into three grades: high-efficiency (AEV > = 0.800), medium-efficiency (0.800 > AEV > = 0.600) and low-efficiency (AEV < 0.600). On this basis, GIS10.2 software is used to draw the spatial distribution map of the allocation efficiency of the rural public health resources in China in 2008, 2011, 2014, and 2017, as shown in Figure 4.

In 2008, the allocation efficiency of the rural public health resources showed an obvious aggregation effect of “the high-efficiency province aggregation and the low-efficiency province aggregation” [40]. In terms of high-efficiency, there are 13 provinces with high-efficiency, including six provinces in the eastern region, three provinces in the central region, and four provinces in the western region. In terms of low-efficiency, there are 14 provinces with low-efficiency, accounting for 45% of 31 provinces. These provinces with low efficiency are mainly concentrated in the central and western regions, including seven provinces in the western region, three provinces in the central region, and four provinces in the eastern region. There are four provinces with medium efficiency, that is, Shanghai, Hubei, Hunan and Yunnan, and their distribution is relatively scattered.

Because of the unbalanced development of China’s economy, the supply of the rural public health resources in different provinces showed an unbalanced state in 2011, and accordingly resulted that the allocation efficiency of the rural public health resources presented an obvious unbalanced trend of “the high-efficiency province reduction, the medium- and low-efficiency province expansion”. The number of the high-efficiency provinces shrank from 13 to six, with Hebei in the eastern region becoming a low-efficiency province and six provinces becoming the medium-efficiency provinces, namely, Zhejiang, Fujian, and Shandong in the eastern region, Anhui and Henan in the central region, and Guangxi in the western region. With this change, the number of the low-efficiency provinces increased to 16 and the number of the medium-efficient provinces increased to 9.

In 2014, because of the implementation of the regional coordinated development strategy, the supply of the rural public health resources tended to balance, and the unbalanced trend of “the high-efficiency province reduction, the medium- and low-efficiency province expansion” presented by the allocation efficiency of the rural public health resources was eased. The number of the low-efficiency provinces had no changes and was still 15. The number of the medium-efficiency provinces shrank to seven. The number of the high-efficiency provinces had an obvious increase, from six to nine.

Although the regional coordinated development strategy has been continuously deepened, the allocation efficiency condition of the rural public health resources in 2017 is the same as that in 2014. In short, the unbalanced problem of the rural public health resource supply is still noticeable. There is a long way to further reform the allocation of the rural public health resources.

### 3.3. Regional Differences in Allocation Efficiency of Rural Public Health Resources in China

In order to further explore the source of the regional differences in the allocation efficiency of the rural public health resources in China, the Theil index model and Gini index model are used to measure the regional differences and their sources in the allocation efficiency of the rural public health resources in China. 

#### 3.3.1. Total Regional Differences in Allocation Efficiency of Rural Public Health Resources in China

**Figure 5:**
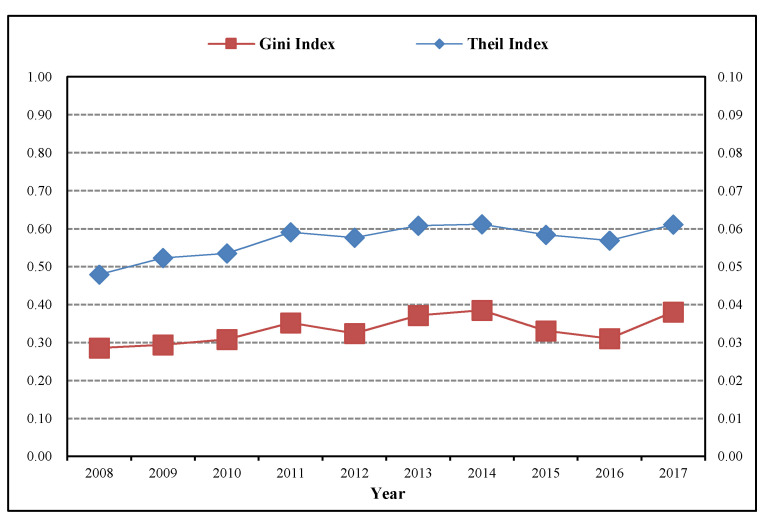
Total Theil index and total Gini index of regional differences in allocation efficiency of rural public health resources in China from 2008 to 2017.

Figure 5 presents the total Theil index and the total Gini index of the regional differences in the allocation efficiency of the rural public health resources in China from 2008 to 2017. The total Theil index is slightly higher than the total Gini index, and they show the same change rule. The regional differences in the allocation efficiency of the rural public health resources in China show an inverted U-shaped development trend, first rising and then falling as a whole. Specifically, the total Theil index of the allocation efficiency of the rural public health resources was the lowest in 2008, and was only 0.0479. Then it was in a rising condition from 2009–2014 and rose to 0.0613 in 2014. This is because China launched the rural medical and health system reform in 2009, but the impact of the financial crisis led to the different promotion speed of the rural medical and health system reform in different provinces, and then resulted in an increasingly expanding total Theil index of the allocation efficiency of the rural public health resources among different provinces. The rural medical and health system reform of different provinces had entered a stable period after 2014, and the policy effect was beginning to gradually appear. The total Theil index of the allocation efficiency of the rural public health resources was tending to shrink and had fallen slightly after 2015, and rose slightly in 2017. Through the comparison, it is found that the changing trend of the intra-regional and inter-regional differences in the allocation efficiency of the rural public health resources are basically consistent with that of the total regional differences.

#### 3.3.2. Sources of Regional Differences in Allocation Efficiency of Rural Public Health Resources in China

Table 2 presents the Theil index decomposition and the Gini index decomposition of the regional differences in the allocation efficiency of the rural public health resources in China from 2008 to 2017. From 2008 to 2017, the average contribution rate of the intra-regional differences measured by the Theil index is 98.67% and much higher than that of the inter-regional differences (1.33%), while the average contribution rate of the intra-regional differences measured by the Gini index is 65.26% and also much higher than that of the inter-regional differences (17.34%). This shows that the intra-regional differences have become the major source of the regional differences in the allocation efficiency of the rural public health resources in China. This is because, since 2008, the Chinese government has attached great importance to the equalization of the inter-regional rural public health resource supply, and has put forward a new round of regional coordinated development policies, such as Western Development, the overall revitalization of the old industrial bases in the northeast China, and the rise of the central China, especially increasing support for the ethnic minority areas, the border areas, and the poor areas, and has fully implemented a series of health poverty alleviation policies. Those play an important role in promoting the optimal allocation of the regional rural public health resources. As the complex natural geographical situation, economic conditions, and social background among provinces within different regions, there is a great difference in the improvement degree of the allocation efficiency of the rural public health resources. The intra-regional differences become the major cause of the regional differences in the allocation efficiency of the rural public health resources in China. China’s economy has gradually recovered from the financial crisis after 2013, but the recovery degree varies in different regions. The economically developed eastern region is recovering faster than the central and western regions. The contribution rate of the inter-regional differences of the allocation efficiency of the rural public health resources had presented a sustained rising state after 2013, and the inter-regional Theil index and Gini index rose to 2.56% and 18.8% separately in 2017. The result shows that if the inter-regional differences in the allocation efficiency of the rural public health resources are allowed to expand, it will not only deviate from the coordinated development goal of the regional rural public health resources, but also increase the difficulty of the coordinated development of the regional rural public health resources.

In addition, the Gini index also provides the specific decomposition of the regional differences in the allocation efficiency of the rural public health resources in China, as shown in Table 3. From the perspective of the inter-regional differences, the differences between the eastern and western region are the largest, followed by the differences between the eastern and central region, and the differences between the central and western region are the smallest. From the perspective of the change rule, with the implementation of strategies such as Western Development and the rise of the central China, the differences between the central and western region are narrowing. However, due to the agglomeration effect and policy advantages of the economic development in the eastern region, the differences between the eastern and central regions and the differences between the eastern and western regions have been maintaining a very high level. From the perspective of the intra-regional differences, because the economic development level and the location characteristics are very similar, the Gini index of the regional differences in the allocation efficiency of the rural public health resource among provinces in the central region is the smallest, and the gap is generally narrow during the research period. There are great differences in each province within the eastern and western regions and their Gini index has been maintaining a very high level, and the gap is generally expanding during the research period.

### 3.4. Influencing Factors of Allocation Efficiency of Rural Public Health Resources in China

Through the calculation result of the allocation efficiency of the rural public health resources in China, it is found that the allocation efficiency of the rural public health resources in China is relatively low and the interregional differences are noticeable. Next, this paper will further study the major factors that affect the change of the allocation efficiency of the rural public health resources in China. Drawing on the research results of the existing literature, this paper indicates that the allocation efficiency of the rural public health resources is mainly affected by the economic and social factors, as follows in detail:

(1) Economic factors. According to the relevant literature, this paper mainly investigates the three economic variables including the economic development level, the living conditions, and the governmental financial support. First, the economic development level is expressed by the per capita GDP (yuan). It is generally believed that the economic development of a region can provide the strong support for the rural public health expenditure. Second, the living conditions are expressed by the per capita disposable income of rural residents (yuan). It is generally believed that the higher the living standard of rural residents, the higher the cognition and demand for the public health resources. Third, the governmental financial support is expressed by the proportion of the public health expenditure to the total fiscal expenditure. It is generally believed that the higher the public health expenditure, the more likely to cause the waste of funds and the lax management, resulting in the low allocation efficiency.
(2) Social factors. According to common practice of the existing literature, the social factors affecting the public health expenditure are mainly considered from four aspects: the population quantity, the population quality, the population structure, and the social support level. First, the population quantity reflects the demand degree for the public health resources, and then affects the governmental public health expenditure and the allocation efficiency of the public health resources. It is measured by the population density index and is expressed by the number of people per square kilometer in the rural areas. Second, the population quality in an area is mainly reflected in the education level of population. The lower the education level of residents, the lower the cognition and demand for the public health resources, resulting in a lower allocation efficiency of the rural public health resources. The education level is concretely expressed by the proportion of illiterate persons to the rural population aged 15 and above. Third, the population structure will affect the demand for the public health resources and the fiscal expenditure. The larger the urban population in a region, the more public health resources need to be invested in cities, and then the supply and management of the rural public health resources are ignored, resulting in the decline of the allocation efficiency. The population structure is measured by the urbanization level and is concretely expressed by the proportion of the urban population to the total population. Fourth, the social support level reflects the major demand groups of the rural public health resources in a region. It is expressed by the proportion of the rural children, youth, and the elderly population to the total population. The higher the social support level, the higher the demand for the rural public health resources, which will lead to the improvement of the allocation efficiency.

Next, this paper takes seven aspects as the influencing factors of the allocation efficiency of the rural public health resources, that is, the economic development level, the living conditions, the governmental financial support, the population density, the education level, the urbanization level, and the social support level. According to the regional classification standard of the eastern, central, and western regions, the target samples are selected to construct a quantitative model between the allocation efficiency and the influencing factors of the rural public health resources, so as to quantify and analyze the influence of each factor on the allocation efficiency of the rural public health resources in China and its three regions.

Because the value range of the allocation efficiency of the rural public health resources is (0, 1], this paper uses the Bootstrap truncated regression model that can minimize the uncertainty of data and the statistical noise to estimate the parameters. Stata16 software is used in the regression process. Through calculation, it can be seen that the R-squared value and the Adj R-squared value of the four models are bigger than 0.6, and the overall goodness of fit of models is good. The estimation results are shown in Table 4.

(1) There are the regional differences in the impact of the economic development level on the allocation efficiency of the rural public health resources. The eastern and western regions have passed the 1% significance test, and the regression coefficient is respectively 0.313 and −0.212. This indicates that the variable promotes the allocation efficiency of the rural public health resources in the eastern region and hinders that in the western region. For the eastern region, the improvement of the economic development level enables more rural residents to enjoy the fruits of the economic development and obtain more public health resources. For the western region, although the economy has developed, the city-centric unbalanced development strategy will make the government invest more resources in the urban development. Not only is the supply of the rural public health resources insufficient, but the allocation efficiency is also low. The nationwide and the central regions have not passed the significance test.
(2) There are the regional differences in the impact of the living conditions on the allocation efficiency of the rural public health resources. The living condition variable in the nationwide, eastern, and western regions has all passed the 5% significance test except for that in the central region, and the regression coefficient is respectively 0.094, −0.138, and 0.283. This indicates that the variable promotes the allocation efficiency of the rural public health resources in the nationwide and western regions, and hinders that in the eastern region. As a developing country, China has a large proportion of rural residents with poor living conditions. With the implementation of the national poverty alleviation strategy, the living conditions of rural residents have been improved and the demand for the public health resources has increased, and then the allocation efficiency of the rural public health resources has been improved. The improvement of the living conditions has greatly increased the demand for the public health resources and has a bigger improvement effect on the allocation efficiency of the rural public health resources, especially in the western region with the relatively low per capita disposable income of rural residents. However, the per capita disposable income of rural residents in the eastern region is very high, and they pay more attention to their own health and are less likely to get sick. The further improvement of the living standards reduces the allocation efficiency of the rural public health resources instead.
(3) The governmental financial support plays an obstacle role in improving the allocation efficiency of the rural public health resources. The coefficient of the governmental financial support variable in the nationwide, eastern, central, and western regions is respectively −0.197, −0.306, −0.004, and −0.098, and all of them have passed the 5% and below significance test except for that in the central region. This shows that with the increase of the total financial inputs into the public health in China, the rural public health expenditure is also increasing year by year. However, the system and mechanism problem of the public health management gives rise to the spatial imbalance of the public health resource supply, and accordingly leads to the mismatch between supply and demand and distorts the allocation efficiency of the rural public health resources.
(4) The population density plays a promotion role in improving the allocation efficiency of the rural public health resources in China. The population density variable in the nationwide, eastern, and western regions has all passed the 5% and below significance test except for that in the central region, and the regression coefficient is respectively 0.065, 0.060, and 0.052. This is mainly because the high population density brings the scale efficiency to the utilization of the rural public health resources, and then improves the allocation efficiency of the rural public health resources.
(5) The education level plays a promotion role in improving the allocation efficiency of the rural public health resources in China. The regression coefficient of the education level variable in the nationwide, eastern, central, and western regions is respectively −0.003, −0.011, −0.018, and −0.013, and all have passed the 10% and below significance test. This indicates that the higher the illiterate person rate in rural residents, the lower the allocation efficiency of the public health resources. With the higher education level of villagers, the greater the demand for the public health resources. This is conducive to the effective allocation of the rural public health resources.
(6) The urbanization level plays an obstacle role in improving the allocation efficiency of the rural public health resources in China. The regression coefficient of the urbanization level variable in the nationwide, eastern, central, and western regions is respectively −0.308, −0.673, −0.122, and −0.375, and all of them have passed the 1% significance test except for that in the central region. When other conditions remain the same, because of the improvement of the urbanization level, a great deal of rural population flows into cities, and the rural public health resources are relatively idle and have not been efficiently utilized.
(7) The social support level plays a promotion role in improving the allocation efficiency of the rural public health resources in China. The regression coefficient of the social support level variable in the nationwide, eastern, central, and western regions is respectively 0.576, 0.355, 0.684 and 0.444, and all have passed the 5% and below significance test. Compared with the middle-aged and young people, the elderly and children have a greater demand for the rural public health resources. Therefore, the higher the rural social support level, the higher the demand for the rural public health resources, and the more fully the rural public health resources may be utilized. And then improve the allocation efficiency of the rural public health resources.

## 4. Conclusions

In this paper, the game competition relationship is included in the evaluation model, and the game cross-efficiency model is used to measure the allocation efficiency of the rural public health resources in 31 provinces of China from 2008 to 2017. Then, the Theil index model and the Gini index model are applied in exploring the regional differences in the allocation efficiency of the rural public health resources and its sources. Finally, the bootstrap truncated regression model is used to analyze the influencing factors of the allocation efficiency of the rural public health resources in China. The major conclusions are as follows:

(1) The total allocation efficiency level of the rural public health resources in China from 2008 to 2017 is relatively low, and it presents a U-shaped trend of first falling and then rising.
(2) The changing trend of the allocation efficiency of the rural public health resources in the eastern, central, and western regions of China from 2008 to 2017 is similar to that in the nationwide region, and it shows a gradient trend that “the allocation efficiency in the eastern region is high, the allocation efficiency in the western region is low, and the allocation efficiency in the central region is at the medium level”. However, the gap among the three regions is continually narrowing.
(3) Because of the unbalanced development of China’s economy, the supply of the rural public health resources in different provinces showed an unbalanced state, and accordingly resulted that the allocation efficiency of the rural public health resources presented an obvious unbalanced trend of “the high-efficiency province reduction, the medium- and low-efficiency province expansion”. With the continuous deepening of the regional coordinated development strategy, the supply of the rural public health resources tended to balance, and the unbalanced trend of the allocation efficiency of the rural public health resources was eased. However, the unbalanced problem of the rural public health resource supply is still noticeable.
(4) To judge from the source of the regional differences, from 2008 to 2017, the average contribution rate of the intra-regional differences measured by the Theil index is 98.67% and much higher than that of the inter-regional differences (1.33%), while the average contribution rate of the intra-regional differences measured by the Gini index is 65.26% and also much higher than that of the inter-regional differences (17.34%). This shows that the intra-regional differences have become the major source of the regional differences in the allocation efficiency of the rural public health resources in China. However, the contribution rate of the inter-regional differences had presented a sustained rising state after 2013, and it cannot be ignored.
(5) The improvement of the education level and the social support level will generally improve the allocation efficiency of the rural public health resources in China and its three regions. The improvement of the governmental financial support and the urbanization level will reduce the allocation efficiency of the rural public health resources in China and its three regions. The economic development level, the living conditions and the population density are the important influencing factors of the allocation efficiency differences of the rural public health resources in the three regions.

The above research results can provide the policy basis for improving the allocation efficiency of the rural public health resources in China.

First, the health poverty alleviation project should be deeply implemented to ensure that the rural poor population enjoys the basic medical and health services, and prevent the poverty caused by diseases. The prices of the rural public health products should be continuously reduced, and the government should provide the corresponding free health preventive services, or subsidize families who take the initiative to take the health preventive services, so that the rural population can get the health preventive services as easily as possible. By constantly perfecting the national poverty alleviation strategy and policy system, the organic connection between the health services and the poverty alleviation can be realized, and the incidence of the rural poverty can be greatly reduced.

Second, the healthy China strategy should be further pushed forward, and more attention should be paid to the improvement of the allocation efficiency on the basis of ensuring the growth of the total supply of the rural public health resources. On one hand, the city-centric supply mode of the public health resources should be changed, the public health resources should be constantly pushed forward to tilt to the rural areas, and the system reform of the new rural cooperative medical insurance should be deepened. The hierarchical diagnosis and treatment system reform of China should be actively pushed forward, and it should be ensured that the high-quality medical resources can enter the rural areas to make the rural residents share the public health and economic development fruits. On the other hand, the system and mechanism reform of the rural public health resource supply should be deepened, restructuring of the rural grass-roots medical institutions should be pushed further forward, and the medical community should be established. A large information sharing platform of the urban and rural medical systems should be established to achieve the continuous records of the electronic health archives and the electronic medical records of the rural residents as well as the information sharing among different levels and types of medical institutions, so as to improve the accessibility of the high-quality medical resources and the total medical service efficiency.

Third, on the basis of continually narrowing the inter-regional differences among the eastern, central, and western regions, more attention should be paid to the intra-regional differences of the allocation efficiency of the rural public health resources among the different provinces. On one hand, the regional rural public health coordinated development strategy should be thoroughly implemented, and the mechanism and system reform of the rural public health resource supply within the region should be coordinated and pushed forward, so as to constantly promote the spatial balanced development of the rural public health resource supply. On the other hand, the regulatory mechanism and the accountability mechanism of the rural public health funds should be established and perfected, and efforts should be made to establish an efficiency-oriented regional rural public health resource supply mechanism, so as to constantly narrow the regional differences in the allocation efficiency of the rural public health resources and realize the effective match for supply and demand of the rural public health resources.

Fourth, various economic and social policies should be constantly optimized to jointly improve the allocation efficiency of the rural public health resources. First of all, each region should increase the investment in the rural education and constantly improve the education level of rural residents, so as to improve their demand for and utilization rate of the rural public health resources. Secondly, each region should follow up and pay attention to the rural unoccupied village phenomenon caused by the improvement of the urbanization level, and duly adjust the layout of the rural public health resource supply, so as to avoid the efficiency loss caused by the idle rural public health resources. Thirdly, the rural revitalization strategy should be accelerated. Each region should promote the transformation of the unoccupied villages into the gathered central villages by the revocation of townships and the merging of villages so as to achieve the scale effect. Finally, the poverty alleviation strategy should be pushed further forward to increase the per capita disposable income of farmers, and the living conditions of the rural residents in the poor areas should be constantly improved so that they can share the high-quality public health resources.

## Figures and Tables

**Table 1:** Evaluation index system of rural public health resource allocation efficiency in China.

Index	Variable	Variable Declaration
input index (x_k0_)	personnel input (x_1_)	number of personnel in rural health institutions (person)
	bed input (x_2_)	number of beds in rural health institutions (unit)
	institutional input (x_3_)	number of rural health institutions (unit)
	expenditure input (x_4_)	rural medical and healthcare expenditure (10,000 yuan)
output index (y_i0_)	hospital business output (y_1,2,3_)	rural diagnosis and treatment person-time (10,000 person-time)
		rural number of people receiving hospitalizations (10,000 person)
		rural average hospitalization days (day)

**Table 2:** Theil index decomposition and Gini index decomposition of regional differences and their sources of allocation efficiency of rural public health resources in China from 2008 to 2017.

Year	Theil Index Decomposition					Gini Index						
	Total Regional Differences	Source of Differences		Contribution Rate (%)		Total G	Source of Differences			Contribution Rate (%)		
		Intra-Regional	Inter-Regional	Intra-Regional	Inter-Regional		*G_w_*	*G_nb_*	*G_t_*	*G_w_*	*G_nb_*	*G_t_*
2008	0.0479	0.0469	0.0010	97.94	2.06	0.2855	0.1841	0.0660	0.0354	64.45	23.11	12.43
2009	0.0523	0.0514	0.0009	98.22	1.78	0.2938	0.1970	0.0446	0.0522	67.04	15.19	17.77
2010	0.0535	0.0531	0.0004	99.23	0.77	0.3083	0.2360	0.0452	0.0271	76.54	14.66	8.80
2011	0.0591	0.0586	0.0005	99.10	0.90	0.3516	0.2458	0.0413	0.0645	69.91	11.75	18.35
2012	0.0576	0.0568	0.0008	98.62	1.38	0.3241	0.1959	0.0482	0.0800	60.46	14.88	24.66
2013	0.0608	0.0603	0.0005	99.25	0.75	0.3714	0.2384	0.0610	0.0720	64.18	16.40	19.41
2014	0.0613	0.0607	0.0005	99.15	0.85	0.3849	0.2401	0.0578	0.0870	62.39	15.02	22.59
2015	0.0584	0.0579	0.0005	99.13	0.87	0.3312	0.1966	0.0661	0.0685	59.37	19.97	20.66
2016	0.0570	0.0561	0.0008	98.58	1.42	0.3103	0.1897	0.0735	0.0471	61.15	23.67	15.18
2017	0.0611	0.0595	0.0016	97.44	2.56	0.3804	0.2551	0.0715	0.0538	67.06	18.80	14.14
Mean	0.0569	0.0561	0.0008	98.67	1.33	0.3342	0.2179	0.0575	0.0588	65.26	17.34	17.40

Note: *G_w_* is the intra-group differences, *G_nb_* is the inter-group differences, and *G_t_* is the differences of the intensity of transvariation; *G = G_w_ + G_nb_ + G_t_*.

**Table 3:** Gini index decomposition of regional differences in allocation efficiency of rural public health resources in China from 2008 to 2017.

Year	Inter-Regional Gini Index			Intra-Regional Gini Index		
	Between Eastern and Central Region	Between Eastern and Western Region	Between Central and Western Region	Eastern Region	Central Region	Western Region
2008	0.3701	0.4401	0.3307	0.3571	0.1656	0.3379
2009	0.3889	0.4586	0.3468	0.3671	0.1258	0.3304
2010	0.3627	0.3965	0.3616	0.3350	0.0939	0.3708
2011	0.4398	0.4759	0.3435	0.3218	0.0895	0.3248
2012	0.3872	0.4196	0.3495	0.2913	0.1492	0.3337
2013	0.4248	0.4784	0.3725	0.3369	0.1497	0.3293
2014	0.4431	0.4501	0.3418	0.3591	0.1299	0.3120
2015	0.3716	0.4440	0.3947	0.3837	0.0943	0.3789
2016	0.3304	0.3907	0.3352	0.3542	0.1134	0.3516
2017	0.4013	0.4614	0.3718	0.3718	0.1345	0.3856

**Table 4:** Bootstrap truncated regression results of influencing factors of allocation efficiency of rural public health resources.

Explanatory Variable	Nationwide	Eastern	Central	Western
economic development level	−0.055 (−1.031)	0.313 *** (3.319)	−0.173 (−0.748)	−0.212 *** (−2.785)
living conditions	0.094 ** (2.135)	−0.138 ** (−2.974)	0.156 (0.868)	0.283 ** (4.926)
governmental financial support	−0.197 *** (−4.713)	−0.306 *** (−3.013)	−0.004(−0.042)	−0.098 ** (−2.138)
population density	0.065 *** (9.318)	0.060 ** (2.048)	−0.049 (−0.514)	0.052 *** (7.832)
education level	−0.003 * (−1.774)	−0.011 * (−1.723)	−0.018 ** (−2.203)	−0.013 *** (−6.248)
urbanization level	−0.308 *** (−3.452)	−0.673 *** (−3.821)	−0.122(−0.254)	−0.375 *** (−3.934)
social support level	0.576 *** (13.354)	0.355 ** (2.643)	0.684 *** (3.657)	0.444 *** (8.543)
constant	−0.244 (−0.863)	0.601 (0.867)	−0.841 (−0.654)	0.323 (1.031)
R-squared	0.684	0.734	0.833	0.884
Adj R-squared	0.676	0.722	0.817	0.876
sample N	310	110	80	120

**Note**: *Z* value is expressed in brackets; * represents 10% significance level, ** represents 5% significance level, *** represents 1% significance level. Bootstrap method is used to set the sample number of 1000 times.
